# Bioprosthesis in aortic valve replacement: long-term inflammatory response and functionality

**DOI:** 10.1136/openhrt-2022-002065

**Published:** 2022-08-04

**Authors:** Huitzilihuitl Saucedo-Orozco, Jesus Vargas-Barron, Ricardo Márquez-Velazco, Julio Iván Farjat-Pasos, Karla Susana Martinez-Zavala, Valentin Jiménez-Rojas, Sergio Andres Criales-Vera, Jose Antonio Arias-Godínez, Giovanni Fuentevilla-Alvarez, Veronica Guarner-Lans, Israel Perez-Torres, Gabriela Melendez-Ramirez, Tomas Efrain Sanchez Perez, Maria Elena Soto

**Affiliations:** 1 Cardioneumology, Instituto Nacional de Cardiologia Ignacio Chavez, Ciudad de Mexico, Mexico; 2 Speciality Hospital, National Medical Center "La Raza", Cardioneumology, Instituto Mexicano del Seguro Social, Ciudad de Mexico, Mexico; 3 Pharmacology, Instituto Nacional de Cardiologia Ignacio Chavez, CDMX, Mexico; 4 Department of Immunology, Instituto Nacional de Cardiologia Ignacio Chavez, Mexico City, Mexico; 5 Interventional Cardiology, Instituto Nacional de Cardiologia Ignacio Chavez, Tlalpan, Mexico; 6 Medicine School, Metropolitan Autonomous University, Ciudad de Mexico, Mexico; 7 Immunology, Instituto Nacional de Cardiologia Ignacio Chavez, CDMX, Mexico; 8 Tomography, Instituto Nacional de Cardiologia Ignacio Chavez, CDMX, Mexico; 9 Echocardiography, Instituto Nacional de Cardiologia Ignacio Chavez, Estado de Mexico, Mexico; 10 Physiology, Instituto Nacional de Cardiologia Ignacio Chavez, CDMX, Mexico; 11 Cardiovascular Biomedicine, Instituto Nacional de Cardiologia Ignacio Chavez, CDMX, Mexico; 12 Magnetic Resonance, Instituto Nacional de Cardiologia Ignacio Chavez, CDMX, Mexico; 13 Applied Biotechnology, Instituto Nacional de Cardiologia Ignacio Chavez, CDMX, Mexico; 14 Cardiovascular Line, Hospital ABC, Mexico City, Mexico

**Keywords:** Echocardiography, Aortic Valve Insufficiency, Aortic Valve Stenosis, Heart Valve Prosthesis

## Abstract

**Background:**

The evaluation of long-term inflammatory response and function in postoperative patients with aortic valve replacement (AVR) deserves special analysis because it is important to try to prevent reoperation and improve durability and functionality of the prostheses. It is our objective

**Methods:**

In this study, we included a cohort of patients with aortic valve damage treated by AVR with mechanical prosthesis, bio prosthesis and we included a control group.

**Results:**

We found that IL-4 and osteopontin levels were higher in patients with mechanical vs biological prostheses (p=0.01 and p=0.04, respectively), osteoprotegerin (OPG) levels were decreased (p=0.01), women had lower levels of ET-1 and IL-6, (p=0.02) (p=0.04), respectively. Patients older than 60 years had decreased levels of IL-1ß p<0.001) and a higher concentration of IL-4 p<0.05). IL-1ß, OPG and TNFα were higher in patients with less than 5 years of evolution vs more than 10 years (p=0.004, p=0.02 and p=0.03, respectively). Factors such as age, gender, prosthetic and elevated IL-1B and ET-1 levels are associated with valve dysfunction prosthetic. These results indicate that the inflammatory involvement present prior to valve replacement may be perpetuated by various factors in the long term.

**Conclusions:**

The findings provide us with the opportunity to effectively treat patients with AVR in the postoperative period, which could prolong the functionality of the bio prostheses.

**Trial registration number:**

NCT04557345.

**WHAT IS ALREADY KNOWN ON THIS TOPIC**

- Inflammatory mechanisms, among other processes, are strongly associated with a higher risk of dysfunction after prosthetic valve implantation.
- The inflammatory process has been little studied and there is no specific therapy during its evolution.
- Inflammation throughout the postvalve implantation and its association with prosthetic dysfunction remains unclear.

**WHAT THIS STUDY ADDS**

- Factors such as age, gender, regardless of the type of prosthesis material, influence the risk of long-term prosthetic dysfunction. There is a significant incident inflammatory state whose type of cytokines are present deserve attention to evaluate mechanisms of action.

**HOW THIS STUDY MIGHT AFFECT RESEARCH, PRACTICE OR POLICY**

- Prevention or delay through anti-inflammatory and specific therapy in subjects undergoing valve implantation independent of age, gender and type of prosthesis could reduce the risk of subsequent dysfunction.
- Clinical trials in this regard and further histological and biomarker studies may be necessary to reduce prosthetic valve dysfunction.

## Introduction

Aortic valve disease affects more than 26% of adult patients over 65 years of age^1^; the main indication for aortic valve replacement (AVR) is aortic stenosis (AS), an active biological process with similarities atherosclerosis.^2^ It begins with a lesion in the valvular endothelium that promotes the accumulation of lipoproteins and infiltration by macrophages and T lymphocytes. These cells secrete tumour growth factor (TGF-α), interleukin 1-β (IL-1β)^3^ and other cytokines that generate the synthesis of matrix metalloproteinases and promote local remodelling.^4^

In parallel, osteogenesis occurs when resident interstitial aortic valve cells (AVICs) are activated to fibroblasts by tumour necrosis factor-alpha (TNF-α) and IL-1β, cells differentiate to fibrotic tissue and osteoblast-like cells. This process is promoted by the action of IL-6 and IL-4,^5^ as well as by other promoter factors such as Osteopontin (OPN),^6^ the osteoprotegerin axis (OPG), the receptor activator of nuclear factor κB (RANK) and its ligand (RANKL).^7^ These processes perpetuate valvular calcification, progressive reduction of the aortic valve area (AVA).

The treatment for severe AS is AVR; however, the inflammatory state will persist in half of the patients in the short term.^8^ Among them are prosthetic material (titanium),^9^ the haemodynamic flow profile, the biological prosthetic valve tissue (porcine or equine) or mechanical factors.^10^ Long-term surgical success can be improved with preoperative and postoperative therapeutic measures.

This study’s objective was to evaluate the function and long-term inflammatory response in postoperated patients with AVR using bioprostheses or mechanical prostheses.

## Methods

A retrospective study in a cohort of postoperated patients with AVR. between January 1995 and January 2020. We included patients older than 18 years of age who met diagnostic criteria for severe valve dysfunction,^11^ and the Heart Team decided to perform AVR using a biological or mechanical prosthesis.^12^ All patients with signed informed consent format. Were excluded Patients with other cardiac intervention in addition to AVR, with significant coronary artery disease, immunodeficiency and oncological or rheumatic disease

We included for the evaluation a control group with 80 healthy subjects matched by age and gender.

We extracted from the clinical record and performed a two-dimensional transthoracic echocardiogram (TTE) before surgery, following the evaluation and recommendations for measuring the heart chambers^13^ to rule out structural abnormalities and valvular dysfunction. Through brachial venipuncture, blood samples were obtained for the quantification of inflammatory mediators. Preoperative and postoperative data were collected.

Enrolment did not imply additional diagnostic procedures to sampling and no alternative or additional treatments were performed. Data management was carried out in order to make identification of an individual patient and the need for consent was required.

### Study design

Outpatient follow-up for valve diseases was performed after AVR for 1–25 years after the intervention. Clinical questioning, physical examination and TTE were performed following the recommendations for the evaluation of valve prostheses.^14^ We obtained Blood samples by brachial venipuncture to quantify biomarkers with a prior signed informed consent form.

### Determination of cytokines

Ten mL of peripheral blood were centrifuged at 2500 rpm for 10 min at 4°C. Then, 500 µL aliquots of serum were prepared and stored at −75°C until cytokine determination. Subsequently, endothelin 1 (ET-1), IL-1ß, IL-1, IL-6, OPN, OPG, RANK, RANK-L and TGF-α were determined by an ELISA in DuoSet sandwich (R&D Systems, Minneapolis, MN), according to the instructions provided by the manufacturer.^15^

### Two-dimensional transthoracic echocardiographic study

TTE was performed and evaluated by two expert echocardiographers following the recommendations for cardiac chamber quantification^13^ and evaluation of valve prostheses.^14^ A Phillips EPIC 7 (Philips, Andover Massachusetts) ultrasound was used with an S5-1 (1–5 MHz) transducer. The effective orifice area (EOA) of the PrAV (aortic valve prosthesis) was calculated employing the continuity equation. The peak velocity (Pvel) and mean Gradient (MG) of the PrVA were obtained in a five-chamber projection. The pulmonary artery (PSAP) was calculated by adding the right atrium pressure to the maximum Gradient of tricuspid regurgitation.

### Type of implanted prosthesis

**Figure 1:**
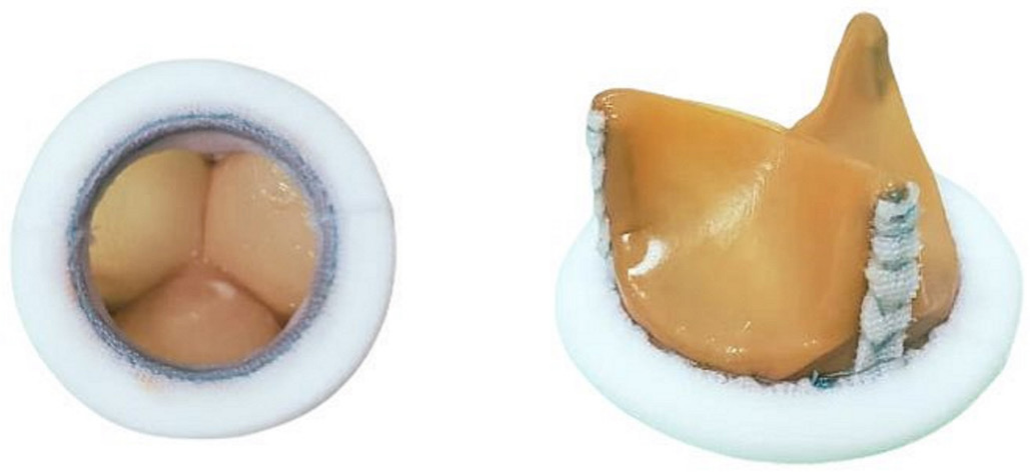
Bioprosthesis INC, manufactured at the ‘Ignacio Chávez’ National Cardiology Institute.

The brands and types of mechanical prostheses placed were St. Jude Masters HP, On-X, Edwards-MIRA, Medtronic-Hall, A.T.S. 3f, Carbomedics Orbis. The bioprostheses placed were St. Jude Epic, Carpentier-Edwards PERIMOUNT and bioprostheses manufactured by the Instituto Nacional de Cardiología ‘Ignacio Chávez’ (figure 1). These prostheses were made of bovine pericardial tissue, with a rigid titanium ring, which has been manufactured since 1976.^16^

### Statistical analysis

Categorical variables were expressed in proportions, and when there was continuity, they were expressed as mean±SD or median and IQR according to the distribution. Comparisons were made using the χ^2^ test or Fisher’s exact test for categorical variables. For dimensional variables, Student’s t-test or Mann-Whitney U tests were applied. Survival analysis was performed using Kaplan-Meier curves. Kruskal-Wallis test and a post hoc analysis using Dunn’s test. Multiple comparison tests were done with adjustment by Bonferroni correction. Differences were considered statistically significant when the p value was <0.05. Statistical analyses were performed using STATA V.16 software.

## Results

### Clinical and demographic characteristics

A total of 156 patients were evaluated, and 76 patients were excluded due to another valvular disease’s coexistence. A total of 80 patients satisfied the inclusion criteria, and their demographic and clinical characteristics are shown in table 1.

**Table 1:** Demographic and clinical characteristics of patients undergoing aortic valve replacement

Variables median (IQR 25–75) n (%)	Total	Biologic prosthesis	Mechanic prosthesis	P value
	n=80	n=53	n=27	
Age, years	56.9 (43.4–65.3)	62.5 (48.9–67.2)	46.7 (31.8–59.8)	0.001*
Male	48 (60)	30 (56.6)	18 (66.6)	0.38†
BSA (m^2^)	1.76±0.17‡	1.74±0.16‡	1.80±0.18‡	0.13§
BMI (kg/m^2^)	27.5 (24.4–29.9)	27.1 (24.2–29.9)	26.8 (25.3–30.8)	0.81*
NYHA class II	27 (33.7)	17 (37)	10 (32)	0.65†
Smoking	14 (17.5)	11 (20.7)	3 (11.1)	0.36¶
Obesity	19 (23.7)	12 (22.6)	7 (25.9)	0.74†
Hypertension	56 (70)	36 (67.9)	20 (74)	0.57
Diabetes	22 (27.5)	15 (28.3)	7 (25.9)	0.82
Dyslipidaemia	40 (50)	24 (45.2)	16 (59.2)	0.23
DAVD	43 (53.7)	31 (58.4)	12 (44.4)	0.23
Statins	39 (48.7)	27 (50.9)	12 (44.4)	0.58
Haemoglobin (g/L)	147(131–156)	145(125–159)	149(136–157)	0.50*
Serum creatinine (mg/dL)	0.8 (0.7–1.0)	0.9 (0.7–1.0)	0.78 (0.6–0.9)	0.02*
eGFR (mL/min/m^2^)	82.9±24‡	78.3±24.1‡	91.9±21.4‡	0.01§
Glucose (mg/dL)	99 (92.8–112.9)	100.8 (93.1–113.5)	96.6 (89.9–108.3)	0.33*
LDL (mg/dL)	112.8 (88–139.5)	107.5 (85.2–130.9)	129 (96.7–155.6)	0.03
HDL (mg/dL)	41.2 (36.8–48.5)	40.8 (36.8–48.5)	43.6 (36.4–48.6)	0.71
Triglycerides (mg/dL)	157 (118.6–197.1)	147(116-182)	163.7 (127–263.7)	0.09
Total cholesterol (mg/dL)	181 (153–202.5)	178.5 (147.8–191.8)	197.2 (162–234.7)	0.02
Albumin (g/dL)	4.19 (4.1–4.3)	4.1 (4–4.3)	4.3 (4.1–4.5)	0.03
Uric acid (mg/dL)	5.82±1.4‡	5.8±1.3‡	5.8±1.6‡	0.85§
ASA	36(45)	35(66)	1 (3.7)	0.00†
OACs	39 (48.7)	12 (22.6)	27(100)	0.00
Sinus rhythm	68(85)	48 (90.5)	20(74)	0.55¶

*Mann-Whitney U test.

†Pearson’s χ^2^ test.

‡Mean(±SD).

§Student’s t-test.

¶Fisher’s exact test.

ASA, acetylsalicylic acid; BMI, body mass index; BSA, body surface area; DAVD, degenerative aortic valve disease; eGFR, estimated glomerular filtration rate; HDL, high-density lipoprotein; LDL, low-density lipoprotein; NYHA, New York Heart Association; OACs, oral anticoagulants.

### Echocardiographic characteristics

Echocardiography findings in patients undergoing AVR are shown in table 2. No statistically significant differences were found in echocardiographic parameters between prostheses; however, we distinguished differences in LV mass, Pvel, MG, EOA, tricuspid annular plane systolic excursion (TAPSE) and PSAP between prosthesis (p<0.001) (table 3). Using Dunn’s posthoc test in pairs with Bonferroni adjustments, a significant difference was found when comparing the control group and patients post-AVR (p<0.001).

**Table 2:** Echocardiography findings and cytokine levels in patients undergoing aortic valve replacement

Variables Median (max - min)	Total	Biological prosthesis	Mechanical prosthesis	P value
	n=80	n=53	n=27	
Echocardiographic findings				
LVEF (%)	60(24-68)	60(24-68)	60(30-68)	0.67*
LV mass (gr/m^2^/BSA)	90.5 ± 24.9†	87.7 ± 25.2†	95.9 ± 23.8†	0.16‡
Peak Velocity (m/s)	2.5 ± 0.71†	2.5 ± 0.68†	2.58 ± 0.79†	0.63‡
Mean Gradient (mm Hg)	16 (17-72)	16 (17-72)	16 (7-49)	0.95
EOA (cm^2^)	1.6 (0.4–2.6)	1.6 (0.4–2.2)	1.6 (0.53–2.6)	0.44
TAPSE (mm)	18 (12-24)	18 (12-24)	19 (14-22)	0.15
PSAP (mm Hg)	32.5 (18–67)	34(21-67)	31(18-52)	0.50
Cytokine levels				
Endothelin 1 (pg/mL)	0.6 (0.08–13.64)	0.60 (0.08–13.64)	0.60 (0.08–11.13)	0.60
IL-1ß (pg/cm^3^)	3 (3–1282.8)	3 (3–649.9	3 (3–1282.8)	0.16
IL-4 (pg/mL)	30 (30–258.4)	30(30-30)	30 (30–258.4)	0.01
IL-6 (pg/mL)	10 (10–56.5)	10(10-10)	10 (10–56.5)	0.16
Osteopontin (pg/mL)	3618±1293†	3413±1362†	4023±1053†	0.04‡
Osteoprogesterin (pg/mL)	1910.3±822.8†	2071±862.9†	1594.7±641.7†	0.01‡
RANK (pg/mL)	50 (50–1990.6)	50 (50–1288.9)	50 (50–1990.6)	0.19
RANK-L (pg/mL)	50 (50–50)	50 (50–50)	50 (50–50)	NS
TNFα (pg/mL)	15 (11.3–527.1)	15 (11.3–388.7)	15 (15–527.1)	0.47

*Mann-Whitney U test.

†Mean(±SD).

‡Student’s t-test.

BSA, body surface area; EOA, effective orifice area; IL, interleukin; LV, left ventricle; LVEF, left ventricular ejection fraction; NS, not significant; PSAP, pulmonary systolic artery pressure; RANK, receptor activator of nuclear-factor kappa B; RANK-L, receptor activator of nuclear-factor kappa B ligand; TAPSE, tricuspid annular plane systolic excursion; TAPSE, Tricuspid annular plane systolic excursion; TNFα, tumour necrosis factor alpha.

**Table 3:** Differences in echocardiographic findings and cytokine levels in biological and mechanical valves and the control group

Variables Median (max–min)	Biological prosthesis INCN	Imported biological prosthesis	Mechanical prosthesis	Control group	P value
	n=39	n=14	n=27	n=80	
Echocardiographic findings					
LVEF (%)	60(24-67)	59.9 (49–68)	60(30-68)	60(54-69)	0.68
LV mass (gr/m^2^/BSA)	87.3±23.8*	89±29.8*	95.9±23.8*	60.1±9.8*	0.001
Peak velocity (m/s)	2.4 (1.1–3.7)	2.45 (1–4.1)	2.5 (2–4.2)	1 (0.7–2.6)	0.001
Mean gradient (mm Hg)	15(11-23)	19.5 (12–22)	16 (11.9–25)	2 (1.6–4.4)	0.001
EOA (cm^2^)	1.6 (0.4–2.2)	1.6 (0.75–1.9)	1.6 (0.53–2.6)	3.2 (2.5–3.9)	0.001
TAPSE (mm)	18 (12–24)	17 (13–20)	19 (14–22)	21 (19–26)	0.001
PSAP (mm Hg)	34 (21-67)	31.4 (23–40)	31 (18-52)	26 (15-40)	0.001
Cytokine levels					
Endothelin 1 (pg/mL)	0.35 (0.08–11.4)	1.3 (0.08–13.6)	0.60 (0.08–3.7)	0.60 (0.8–2.3)	0.42
IL-1ß (pg/cm^3^)	3 (3–649.9)	3 (3–3)	3 (3–1282.8)	15(15-190)	0.001
IL-4 (pg/mL)	30(30-30)	30(30-30)	30 (30–258.4)	15 (15–141.5)	0.001
IL-6 (pg/mL)	10(10–10)	10(10–10)	10 (10–56.5)	10 (10–195.8)	0.10
RANK (pg/mL)	50 (50–1288.9)	50(50–50)	50 (50–1990.6)	50 (50–409.4)	0.35
RANK-L (pg/mL)	50 (50–50)	50 (50-50)	50 (50-50)	50 (50–1176)	0.16
TNFα (pg/mL)	15 (15–388.7)	15 (11.3–19.3)	15 (15–527.1)	15 (15–812.6)	0.54
Osteopontin (pg/mL)	3246.5 ± 1369.8*	4238.5 ± 1249.8*	4319.5 ± 1053.9*	2181.6 ± 2247.5*	0.01
Osteoprogesterin (pg/mL)	1928 ± 835.6*	2113.8 ± 962.1*	1376.8 ± 641.7*	838.4 ± 710.2*	0.001

*Mean (±SD), Kruskal-Wallis test, and a post hoc analysis using Dunn’s multiple comparison test with adjustment using Bonferroni or Benjamini-Hochberg corrections.

BSA, body surface area; EOA, effective orifice area; IL, interleukin; LV, left ventricle; LVEF, left ventricular ejection fraction; NIC, National Institute of Cardiology; PSAP, pulmonary systolic artery pressure; RANK, receptor activator of nuclear-factor kappa B; RANK-L, receptor activator of nuclear-factor kappa B ligand; TAPSE, tricuspid annular plane systolic excursion; test settings, comparison test with adjustment using Bonferroni or Benjamini-Hochberg corrections.; TNFα, tumour necrosis factor alpha.

### Measurement of cytokines in valve prostheses

Cytokine levels are shown in table 2. In the analysis by gender and age, women had lower levels of ET-1 than men (0.53 vs 0.67 pg/mL, p=0.02), and there was a difference in IL-6 (p=0.04). Patients older than 60 years had decreased levels of IL-1ß (3–15 pg/cm^3^, p<0.001) and higher concentration of IL-4 (30–15 pg/mL, p<0.05). Differences in cytokine levels in biological and mechanical valves and the control group are shown in table 3.

When comparing by the time of evolution dividing the patients in under 5 years, between 5 and 9 years or patients with more than ten years of placement of the PrAV, and after having performed the AVR, there was no difference in the ET-1 level (p=0.81). IL-1ß, OPG and TNFα were higher in patients with less than 5 years of evolution versus those with more than ten years (p=0.004, p=0.02 and p=0.03, respectively) in the post hoc analysis test.

### Evaluation of prosthetic functionality

Prosthetic valve dysfunction occurred in 18 patients (22.5%), without a difference between patients treated with biological vs mechanical valve prostheses (p=0.96). The main complications were anterior paravalvular leak with a frequency of (15%).

**Figure 2:**
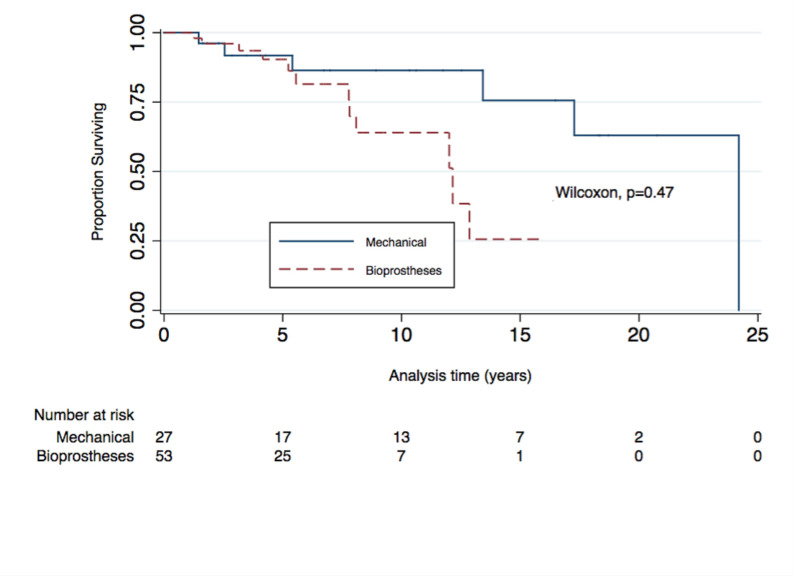
The Kaplan-Meier survival estimates did not show significant differences between patients with mechanical aortic valve and bioprosthetic valve.

The proportion of prosthetic valve dysfunction at 12 years after AVR is 75% for patients with bioprostheses (95% CI=0.49 to 0.93) and 50% for mechanical prostheses (95% CI=0.19 to 0.88). The Wilcoxon test did not show statistical significance between the percentages of prosthetic dysfunction over time (p=0.47).(Figure 2) The restricted mean prosthetic dysfunction was 11.4 years for patients with biological prostheses and 19.4 years for mechanical prostheses. The Cox proportional hazards analysis in post-AVR patients is shown in table 4.

**Table 4:** A Cox proportional hazards regression model stratified by time of follow-up showing the effect of variables on the risk of prosthetic valve dysfunction

Variable	Coefficient (ß)	SE	Wald χ^2^	HR	95% CI	P value
Age over 60 years	−0.97	0.63	−1.55	0.37	0.06 to 2.11	0.26
Biological prosthesis	0.11	0.23	0.17	1.12	0.10 to 1.29	0.86
Endothelin 1 (pg/mL)	0.17	0.10	1.97	1.19	1.0 to 1.41	0.04
IL-1ß (pg/cm^3^)	0.004	0.002	2.11	1.004	1.003 to 1.004	0.035
IL-4 (pg/mL)	−0.03	0.14	−2.07	0.96	0.94 to 0.99	0.039

IL, interleukin.

Prosthetic valve dysfunction is shown in tables 5 and 6. Patients who were implanted with locally manufactured bioprostheses (INC valve) did not show differences in cytokine levels, echocardiographic parameters or prosthetic valve dysfunction than imported bioprostheses (tables 5 and 7). Figure 2 shows survival of functionality and we included a online supplemental graphical abstract.

10.1136/openhrt-2022-002065.supp1 Supplementary data

**Table 5:** Cytokine levels and echocardiographic findings in patients with prosthetic valve dysfunction

Variables Median (max–min)	Bioprosthetic valve dysfunction (n=12)	Mechanical valve dysfunction (n=6)	P value
Echocardiographic findings			
LVEF (%)	60 (24–65)	60 (52–65)	0.96†
LV mass (gr/m^2^/BSA)	90.9±18.6*	108.9±20.7*	0.08‡
Peak Vel (m/s)	2.9 (1.3–3.7)	3.5 (2.2–3.8)	0.054
Mean gradient (mm Hg)	24.5 (10–72)	32.5 (18–35)	0.32
EOA (cm^2^)	1.4 (0.4–2.1)	1 (0.5–2.6)	0.81
TAPSE (mm)	18 (15–20)	21 (17–22)	0.02
PSAP (mm Hg)	38.5 (22–67)	35 (23–47)	0.74
Cytokine levels			
Endothelin 1 (pg/mL)	0.46 (0.08–13.6)	3.1 (0.1–4.3)	0.15
IL-1ß (pg/cm^3^)	3 (3–649.9)	3 (3–513.9)	NS
IL-4 (pg/mL)	30 (30–30)	30 (30–143.1)	0.15
IL-6 (pg/ml)	10 (10–10)	10 (10–10)	NS
Osteopontin (pg/mL)	3453.4 ± 1315.1*	4083 ± 974.1*	0.31*
Osteoprogesterin (pg/mL)	2082.8 ± 861.5*	1768 ± 616.8*	0.43
RANK (pg/mL)	50 (50–1288.9)	50 (50–840.1)	NS
RANK-L (pg/mL)	50 (50–50)	50 (50–50)	NS
TNFα (pg/mL)	15 (15–388.7)	15 (15–461.9)	0.54

*Mean(±SD)

†Mann-Whitney U test.

‡Student’s t-test.

BSA, body surface area; EOA, effective orifice area; IL, interleukin; LV, left ventricle; LVEF, left ventricular ejection fraction; NS, not significant; PSAP, pulmonary systolic artery pressure; RANK, receptor activator of nuclear-factor kappa B; RANK-L, receptor activator of nuclear-factor kappa B ligand; TAPSE, tricuspid annular plane systolic excursion; TNFα, tumour necrosis factor alpha.

**Table 6:** Cytokine() levels and echocardiographic findings in patients with prosthetic valve dysfunction

Variables Median (max–min)	Biological prosthesis		P value	Mechanical prosthesis		P value
	With dysfunction (n=12)	Without dysfunction (n=41)		With dysfunction (n=6)	Without dysfunction (n=21)	
Echocardiographic findings						
LVEF (%)	60 (24–65)	60 (50–68)	NS	60 (52–65)	58 (30–68)	0.61*
LV mass (gr/m^2^/BSA)	90.9±18.6†	86.8±27†	0.62	108.9±20.7†	92.2±23.7†	0.13‡
Peak Vel (m/s)	2.9 (1.3–3.7)	2.4 (1–3.5)	0.03	3.5 (2.2–3.8)	2.4 (1–4.2)	0.00
Mean gradient (mm Hg)	24.5 (10–72)	14 (7–42)	0.00	32.5 (18–35)	15 (7–49)	0.00
EOA (cm^2^)	1.4 (0.4–2.1)	1.6 (1.0–2.2)	0.02	1 (0.5–2.6)	1.8 (1.3–2.1)	0.04
TAPSE (mm)	18 (15–20)	18 (12–24)	NS	21 (17–22)	19 (14–22)	0.04
PSAP (mm Hg)	38.5 (22–67)	32 (21–65)	0.17	35 (23–47)	31 (18–52)	0.52
Cytokine levels						
Endothelin 1 (pg/ml)	0.46 (0.08–13.6)	0.60 (0.08–11.4)	0.87	3.1 (0.1–4.3)	0.18 (0.08–11.3)	0.09
IL-1ß (pg/cm^3^)	3 (3–649.9)	3 (3–75.7)	0.056	3 (3–513.9)	3 (3–1282.8)	0.92
IL-4 (pg/mL)	30 (30–30)	30 (30–30)	NS	30 (30–143.1)	30 (30–250.4)	0.70
IL-6 (pg/mL)	10 (10–10)	10 (10–10)	NS	10 (10–10)	10 (10–56.5)	0.59
Osteopontin (pg/mL)	3453.4±1315.1†	3401.7±1392.4†	0.90	4083±974.1†	4003.3±1097.9†	0.87‡
Osteoprogesterin (pg/mL)	2082.8±861.5†	2067.6±874†	0.95	1768±616.8†	1545.1±654.7†	0.46‡
RANK (pg/mL)	50 (50–1288.9)	50 (50-50)	0.00	50 (50–840.1)	50 (50–1990.6)	0.63
RANK-L (pg/mL)	50 (50–50)	50 (50–50)	NS	50 (50–50)	50 (50–50)	NS
TNFα (pg/mL)	15 (15–388.7)	15 (11.3–305.53)	0.95	15 (15–461.9)	15 (15–527.1)	0.85

*Mann-Whitney U test.

†mean(±SD).

‡Student’s t-test.

BSA, body surface area; EOA, effective orifice area; IL, interleukin; LV, left ventricle; LVEF, left ventricular ejection fraction; PSAP, pulmonary systolic artery pressure; RANK, receptor activator of nuclear-factor kappa B; RANK-L, receptor activator of nuclear-factor kappa B ligand; TAPSE, tricuspid annular plane systolic excursion; TAPSE, Tricuspid annular plane systolic excursion; TNFα, tumour necrosis factor alpha.

**Table 7:** Differences in echocardiographic findings and levels of cytokines in different biological valves

Variables Median (max–min)	Biological prosthesis INC	Imported biological prosthesis	P value
	n=9	n=3	
Echocardiographic findings			
LVEF (%)	60 (24–65)	54 (49–61)	0.25
LV mass (gr/m^2^/BSA)	91.12±13*	90.5±35**	0.96†
Peak velocity (m/s)	3 (2.2–3.7)	2.8 (1.3–3.0)	0.30
Mean gradient (mm Hg)	25 (10–44)	21 (12–72)	0.85
EOA (cm^2^)	1.4 (0.4–2.1)	1.4 (0.7–1.7)	0.78
TAPSE (mm)	18 (16–20)	18 (15–19)	0.62
PSAP (mm Hg)	41 (22–67)	29 (23–37)	0.11
Cytokine levels			
Endothelin 1 (pg/mL)	0.90 (0.08–6.5)	3.0 (0.15–13.6)	0.10
IL-1ß (pg/cm^3^)	3 (3–649.9)	3 (3–3)	0.39
IL-4 (pg/mL)	30 (30–30)	30 (30–30)	NS
IL-6 (pg/mL)	10 (10–10)	10 (10–10)	NS
RANK (pg/mL)	50 (50–1288.9)	50 (50–50)	0.39
RANK-L (pg/mL)	50 (50–50)	50 (50–50)	NS
TNFα (pg/mL)	15 (15–388.7)	15 (15–15)	0.56
Osteopontin (pg/mL)	3358.9±1254.8*	3737±1749.6*	0.68†
Osteoprogesterin (pg/mL)	2011.6±902.1*	2296.5±857.8*	0.64†

*Mean(±SD), Mann-Whitney U test.

†Student’s t-test.

BSA, body surface area; EOA, effective orifice area; IL, interleukin; LV, left ventricle; LVEF, left ventricular ejection fraction; NCI, National Institute of Cardiology; NS, not significant; PSAP, pulmonary systolic artery pressure; RANK, receptor activator of nuclear-factor kappa B; RANK-L, receptor activator of nuclear-factor kappa B ligand; Statistical test, †Student’s t-test.; Statistical test, Mann–-Whitney U test.; TAPSE, tricuspid annular plane systolic excursion; TNFα, tumour necrosis factor alpha.

## Discussion

Calcification of the native aortic valve is present even after AVR. The pathogenesis is multifactorial; factors such as mechanical forces lead to endothelial dysfunction,^2^ altered flow dynamics,^17^ production of inflammatory cytokines by the prosthetic material (titanium)^9^ and xenoantigens such as Galα3Gal and the corresponding anti-Gal antibodies contributing to valve damage. The participation of cytokines before AVR and after the intervention has been studied to define whether this inflammatory damage requires timely therapy to prolong prosthetic durability.^8^

Among the cytokines studied, IL-4 activates collagen synthesis, promotes fibrosis progression, and inhibits inflammatory cytokines production.^18^ Its secretion occurs in response to microorganisms, prosthetic material, volumetric or pressure overload.^19^ In this study, elevated levels of IL-4 were found in patients treated with a mechanical prosthetic implant; however, this increase was not associated with prosthetic dysfunction, rheumatic heart disease, gender or the time to progress after PrVA placement. The elevation of IL-4 could be associated with the inflammation that occurs postsurgery, promoting the alternative activation of macrophages into M2 cells, increasing repair macrophages (M2), and decreasing when interacting with IL-10 and TGF-β. These changes contribute to valve tissue repair, and our results confirm this judgement.

We further found an increase in OPN in patients that received mechanical and biological valve prostheses without statistically significant difference. However, this finding had only been demonstrated in dysfunctional biological prostheses.^20^ One explanation for this finding is the evidence that in calcified porcine aortic valves, there is an increase in OPN, which activates osteogenic signalling.^21^

We found low levels of OPG in mechanical prostheses; in relation, this finding has been found that low levels of OPG lead to an osteoclastic transformation of the valve,^22^ and on the other hand, there is a negative correlation between native AVA and OPG.^23^

Increased ET-1 and endothelin receptor-A levels have been identified in patients with native AS,^24^ and endothelin A and B receptors are located on the leaflets’ tips and surface.^25^ There is a transient increase following myocardial damage after AVR and a concomitant diastolic dysfunction^26^; however, this does not persist, and it decreases in conjunction with brain natriuretic peptide after improvement of the ventricular function due to decreased LV afterload.^27^ In our work, the ET-1 level was similar between mechanical or non-functional biological prostheses. However, in mechanical prostheses with prosthetic dysfunction and the first 5 years after AVR, ET-1 was found to increase; this finding is like to previous studies in dysfunctional biological valves.^20^

IL-1β induces inflammation through the inhibition of factor-κβ and AVICs^28^; therefore, its participation in the extracellular matrix remodelling will condition the proliferation of interstitial cells and the expression of MPPs,^3^ and also a dysfunction has also been found in the anti-inflammatory mechanism of the interleukin receptor antagonist 1β. In this research, the levels of IL-1β were similar in patients receiving biological and mechanical prostheses with and without dysfunction. However, in dysfunctional prostheses to a long-term time (more than 10 years), there was a decrease in IL-1β, which suggests that its participation is broad and varies according to comorbidities, the prosthesis material gender, and time of evolution.

Before AVR, the inflammatory process will continue and persist; however, anti-inflammatory therapy should be proposed after implantation. The transformation process of AIVCs leads to postoperative valve dysfunction since they change to a myofibroblast phenotype that is activated in the presence of transforming growth factor beta1 (TGF-β1).^29^ Recently proposed therapies such as l-arginine prevent osteogenic differentiation of AVICs and reduce matrix calcification regarding therapeutics. This effect is obtained through the modulation of proteins involved in the cellular redox system, the extracellular matrix’s remodelling, and the inflammatory activation of AVICs.^30^

Prostheses’ advantages and disadvantages are well defined, including inflammation in the early and late postoperative periods. Studies that include punctual monitoring still require exploration through systematic randomised clinical trials to improve valve prostheses’ functionality.

## Conclusions

The inflammatory process present after AVR is chronic and multifactorial. OPN and ET-1 are increased in mechanical prostheses. There are no differences in durability and prosthetic dysfunction; however, the increase in IL-1β and ET-1 is associated with a greater risk of prosthetic valve dysfunction regardless of prosthetic valve type. Factors such as age, gender, inflammatory status and type of prosthetic material influence long-term dysfunction.

10.1136/openhrt-2022-002065.supp2 Abstract translation This web only file has been produced by the BMJ Publishing Group from an electronic file supplied by the author(s) and has not been edited for content.

## Data Availability

Data are available on reasonable request. The data are available on reasonable request to the corresponding author.
