# Supplementary figures and images for: Bioprosthesis in aortic valve replacement: long-term inflammatory response and functionality
Source: Open Heart. 2022 Aug 4;9(2):e002065. doi: 10.1136/openhrt-2022-002065 (PMC9358956; doi:10.1136/openhrt-2022-002065)

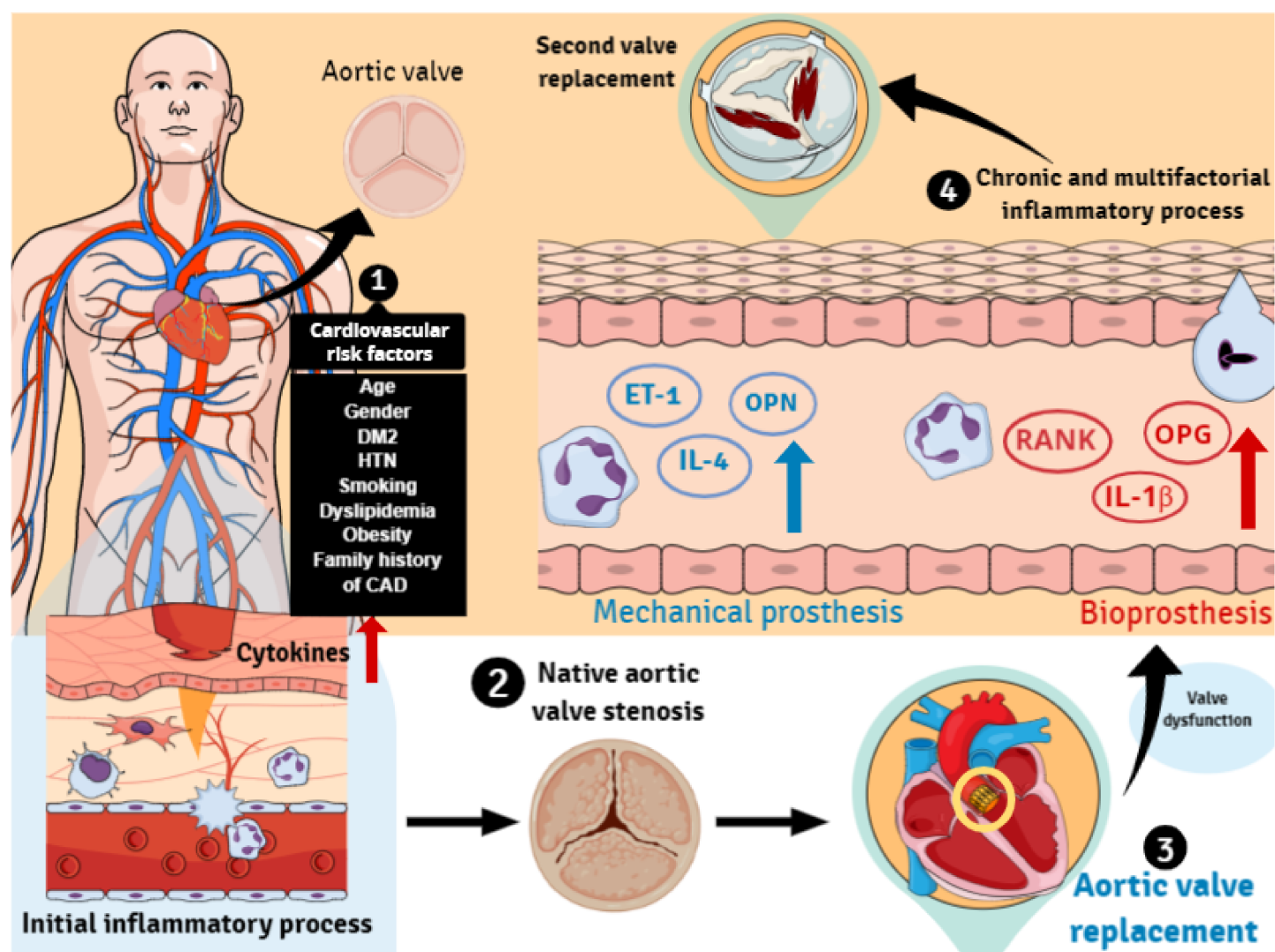

Supplement: Supplementary data [file openhrt-2022-002065supp001.pdf]
