# Supplementary material for: Bioprosthesis in aortic valve replacement: long-term inflammatory response and functionality
Source: Open Heart. 2022 Aug 4;9(2):e002065. doi: 10.1136/openhrt-2022-002065 (PMC9358956; doi:10.1136/openhrt-2022-002065)
Supplement: Abstract translation [file openhrt-2022-002065supp002.pdf]

La evaluación de la función y respuesta inflamatoria a largo plazo en pacientes postoperados con reemplazo valvular aórtico (RVA) merece un análisis especial porque es importante tratar de prevenir la reintervención y mejorar la durabilidad y funcionalidad de las prótesis. Métodos En este estudio incluimos una cohorte de pacientes con daño valvular aórtico tratados mediante AVR con prótesis mecánica, bioprótesis e incluimos un grupo control. Resultados. Encontramos que los niveles de IL-4 y OPN eran mayores en pacientes con prótesis mecánicas vs biológicas (p = 0,01 y p = 0,04 respectivamente), los niveles de OPG estaban disminuidos (p = 0,01), las mujeres tenían niveles más bajos de ET-1 e IL -6, (p = 0,02) (p = 0,04) respectivamente. Los pacientes mayores de 60 años tenían niveles disminuidos de IL-1ß p <0,001) y mayor concentración de IL-4 p <0,05). IL-1ß, OPG y TNFα fueron mayores en pacientes con menos de 5 años de evolución frente a más de 10 años (p = 0,004, p = 0,02 y p = 0,03, respectivamente). Factores como la edad, el género, la prótesis y los niveles elevados de IL-1B y ET-1 están asociados con la disfunción de la válvula protésica. Estos resultados indican que el compromiso inflamatorio previo al reemplazo valvular puede perpetuarse por varios factores a largo plazo. Conclusiones Los hallazgos nos brindan la oportunidad de tratar de manera efectiva a los pacientes con AVR en el postoperatorio, lo que podría prolongar la funcionalidad de las bioprótesis.
